# Health's role in achieving Australia's Sustainable Development Goal commitments

**DOI:** 10.5694/mja2.50040

**Published:** 2019-02-24

**Authors:** Claire E Brolan, Nina Hall, Sandra Creamer, Ingrid Johnston, Jaya AR Dantas

**Affiliations:** ^1^ Centre for Policy Futures University of Queensland Brisbane QLD; ^2^ Queensland Centre for Intellectual and Developmental Disabilities University of Queensland Brisbane QLD; ^3^ University of Queensland Brisbane QLD; ^4^ National Aboriginal and Torres Strait Islander Women's Alliance Canberra ACT; ^5^ Public Health Association of Australia Canberra ACT; ^6^ Curtin University Perth WA

**Keywords:** Climate change, Public health, International cooperation, Health policy, Social determinants of health, Healthcare disparities

Australia's implementation of the Sustainable Development Goals must advance the nation's health and wellbeing transparently and inclusively

In 2015, along with 192 United Nations member states, Australia adopted the 2030 Agenda for Sustainable Development.^1^ The 17 Sustainable Development Goals (SDGs) that form the basis of this Agenda are “an urgent call for action by all countries — developed and developing — in a global partnership. They recognize that ending poverty and other deprivations must go hand‐in‐hand with strategies that improve health and education, reduce inequality, and spur economic growth — all while tackling climate change and working to preserve our oceans and forests”.^2^

This statement makes it clear: the 17 SDGs are an urgent call to action aimed at all countries, including high income nations such as Australia, to advance planetary health for intergenerational equity and wellbeing (Box 1).^3^, ^4^

**Box 1:**
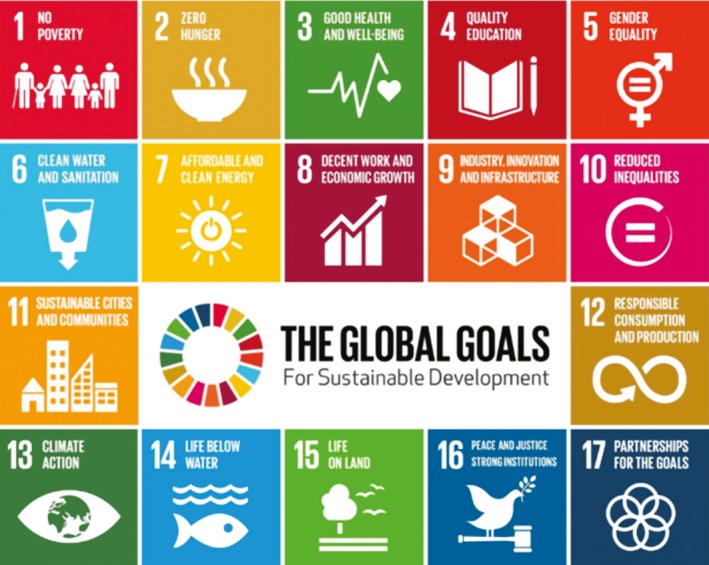
The 17 Sustainable Development Goals that Australia has committed to implement at home and as part of its international development and humanitarian assistance program

The 17 SDGs and 169 associated targets are ambitious and demonstrate the need by governments to continue the work commenced during the Millennium Development Goal (MDG) era. The SDG health goal (ie, SDG 3, ensure healthy lives and promote wellbeing for all at all ages) certainly continues the “unfinished business” of the MDG health agenda (ie, MDG 4, reduce child mortality; MDG 5, improve maternal mortality; and MDG 6, combat human immunodeficiency virus infection and acquired immunodeficiency syndrome, malaria and other diseases). It also tasks countries with addressing their non‐communicable disease and injury burdens, including promoting mental health and strengthening the prevention and treatment of substance misuse, alongside improving access to medicines and to sexual and reproductive health and quality health care services (Box 2).^1^

**Box 2:**
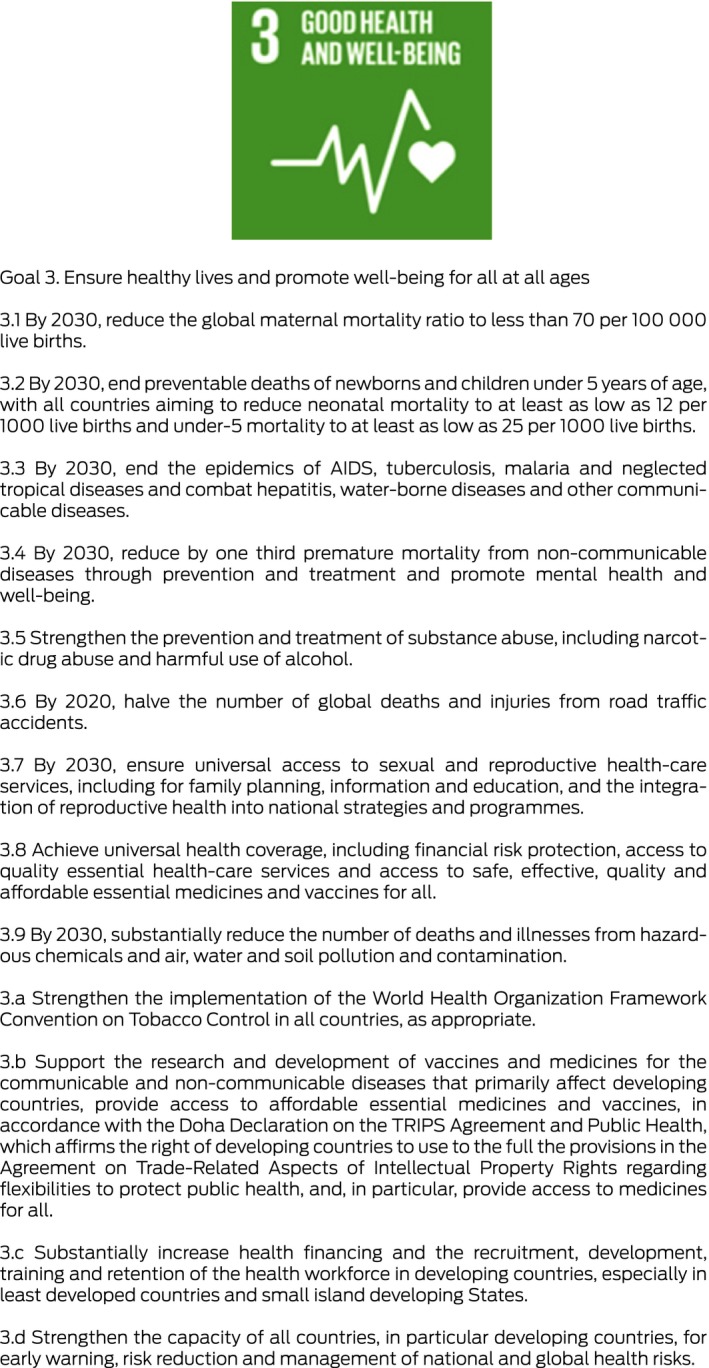
Sustainable Development Goal 3 targets and means of implementation^1^ TRIPS = Trade‐Related Aspects of Intellectual Property Rights. ◆

While there is one SDG explicitly dedicated to health, achieving the remaining SDGs for health is equally crucial (Supporting Information , figure 1).^5^ Indeed, the Australian Department of Health acknowledges that good health is both a precondition and an outcome of the 2030 Agenda.^6^ The SDGs promote social determinants of health such as nutrition (SDG 2), education (SDG 4), gender (SDG 5), water and sanitation (SDG 6), employment (SDG 8), reducing inequalities (SDG 10), housing (SDG 11) and healthy environments (SDGs 13–15). SDGs 16 and 17 further support good governance and multistakeholder partnerships for health, strong data and information systems, and equitable access to quality health care services and associated entitlements. The importance of SDGs 16 and 17 cannot be understated: robust health systems that can generate reliable vital statistics data are key for strengthening the evidence base for SDG policy and planning, critical for improving the health and wellbeing of all Australians.^7^

## Australia's voluntary national review on the Sustainable Development Goals and health

In July 2018, Australia was one of 47 nations to present its voluntary national review (VNR) on achieving the SDGs at the High‐Level Political Forum on Sustainable Development in New York.^8^ Australia's VNR included a four‐page narrative on SDG 3 that provided Australia's medical and public health community with its first real insight into the federal government's position around national SDG priorities. This was a significant step as it has been almost 3 years since Australia agreed to integrate the SDGs into development planning nationally and as part of its Overseas Development Aid strategy.^9^ The Australian Government progress has been marginal; until the recent release of Australia's VNR, the non‐government and corporate sectors had been the main stakeholders promoting Australia's SDG commitments (Supporting Information , table 1).

Although Australia's VNR belatedly signalled to the world the seriousness of the government's approach to the implementation of the 2030 Agenda, it nonetheless says very little around national plans for SDG 3 achievement. The VNR broadly outlines the government plans to leverage the nation's health goal commitments for increased access to a quality and effective health care system (namely universal health coverage).^8^ This includes an impetus on chronic disease prevention and mental health supports, as well as improving the health care of regional, rural and remote Australians, Aboriginal and Torres Strait Islander peoples, and lesbian, gay, bisexual, transgender, intersex and queer (LGBTIQ+) community members (Supporting Information , table 2). The VNR goes on to note that “innovation and technology may assist” in achieving SDG 3, especially in terms of addressing the health needs of people in rural and remote areas by greater integration of digital technology.^8^ However, a cogent SDG program for this integration is not presented in the VNR.

The VNR also states that governments in Australia “recognise the importance of healthy ecosystems and socio‐economic factors to human health, with an interlinked, holistic approach that focuses on the underlying determinants of health, consistent with linkages between SDG 3 and many of the other SDGs”.^8^ However, there is no comment as to how other SDGs that include the social determinants of health will be linked and tracked against Australia's SDG 3 priorities. Again, as inequities associated with the social determinants disproportionately affect Australia's Indigenous peoples and other marginalised populations (eg, persons with disabilities, migrants and refugees, single men and women of all ages), a multidimensional and comprehensive approach to SDG 3 measurement and monitoring by the Australian Government will be imperative.^10^ However, it is regrettable that this was not made explicit in the VNR content.

Certainly, what is most telling about the VNR's brief narrative on Australia's SDG health priorities is what it does not say. The VNR implicitly refers to Australia's commitments under SDG 3 Target 4 (reducing non‐communicable diseases) and SDG 3 Target 8 (achieving universal health coverage), yet it fails to acknowledge how SDG 3 Target 1 (maternal mortality), SDG 3 Target 2 (newborn and child mortality), SDG 3 Target 3 (epidemics and communicable diseases) and SDG 3 Target 7 (access to sexual and reproductive health care services) will be addressed in the Australian context.^8^ The population health priorities of SDG 3 Targets 1, 2, 3 and 7 disproportionately affect some of Australia's most disenfranchised populations, and their overt achievement should be outlined by the Australian Government. The need to leverage all SDG 3 targets (and the broader SDG framework) is especially important to meaningfully value and address, as a priority, the wellbeing of Australia's Aboriginal and Torres Strait Islander peoples.^11^, ^12^, ^13^, ^14^

## Recommendations for the 2030 Agenda health goal implementation in Australia

Australia's first VNR on the 2030 Agenda is not a national report card on SDG implementation. Rather, it is an expression of intent of the federal government's SDG commitment. The Australian Government's ability to substantively report on SDG implementation (including SDG health‐related implementation) is hindered by the country lacking a comprehensive SDG action plan. We recommend the SDG targets and indicators for health should be grounded in a roadmap for action and contextualised to the Australian setting. They need to identify and speak to Australia's unique health landscape and cross‐cutting inequities, and structural and data challenges. An Australian SDG roadmap for health, located in a broader national SDG implementation plan, will also need input, monitoring and review by a formal national advisory body that represents the breadth of Australia's public health sector, private industry, and civil society and community stakeholders. The Australasian College for Emergency Medicine's integration of community representatives to ensure balanced policy decisions is a potential SDG participatory governance model (https://acem.org.au).

The Department of Foreign Affairs and Trade and the Department of the Prime Minister and Cabinet are currently leading an interdepartmental senior officials group to progress whole‐of‐government coordination on how to give effect to the 2030 Agenda in Australia.^15^ This initiative means that responsibilities for reporting on the SDGs are divided across government departments, embedding an ad hoc approach to SDG planning and integration, “antithetical to the SDG vision for game‐changing and innovative development solutions through country leadership, national policy coherence and participatory governance”.^16^ We recommend a single point of government responsibility. France and Belgium have sustainable development ministries tasked with steering whole‐of‐government SDG rollout.

Under Australia's more piecemeal SDG governance arrangement and approach, the Department of Health is tasked with reporting on SDG 3. The Department of Health is also tasked with supporting the Department of Agriculture and Water Resources to implement SDG 2 (zero hunger) (Supporting Information , table 3^15^). It is not known at present whether the Department of Health interfaces with other federal agencies responsible for the social determinants beyond SDG 2, or with Indigenous Affairs or the Office for Women that both sit within the Department of the Prime Minister and Cabinet. It is also unclear whether the Department of Health is responsible for, or even included in, SDG 3‐related regional and global health planning, or whether this alone falls under the SDG remit of the Department of Foreign Affairs and Trade.

The SDG Index and Dashboards, which report on worldwide SDG progress, show SDG 3 to already be an Australian success story.^17^ At a population level that may be so, but this defeats the point of SDG adoption and integration by high income nations such as Australia, given that internationally oriented SDG data reporting platforms are not designed to capture hidden subnational complexities and should be treated with caution. Coupled with a weak VNR, such data platforms have the potential to obscure and mask the intersectional disadvantage and complex health disparities in this country. Therefore, we urge the Australian Government to ensure that Australia's next VNR does not inadvertently perpetuate the invisibility of disadvantaged groups in the Australian community who are at the heart of SDG ambition and its catchcry, “leave no one behind”.^1^ Comprehensive interdepartmental partnership and national policy and planning for SDG coherence, integration, implementation and review, combined with strong centralised governance, social accountability oversight mechanisms and bipartisan political support, will be crucial.

## Competing interests

No relevant disclosures.

## Provenance

Not commissioned; externally peer reviewed.

## References

[1] United Nations General Assembly . Transforming our world: the 2030 Agenda for Sustainable Development (A/RES/70/1). Resolution adopted by the General Assembly on 25 September 2015. http://www.un.org/ga/search/view_doc.asp?symbol=A/RES/70/1&Lang=E (viewed July 2018).

[2] United Nations, Sustainable Development Goals Knowledge Platform. Sustainable Development Goals. https://sustainabledevelopment.un.org/sdgs (viewed Nov. 2018).

[3] Organisation for Economic Co‐operation and Development . Better policies for 2030: an OECD action plan on the Sustainable Development Goals. OECD; 2016 https://www.oecd.org/dac/Better%20Policies%20for%202030.pdf (viewed July 2018).

[4] Clark H . Governance for planetary health and sustainable development. Lancet 2015; 386: e39–e41. 26188750 10.1016/S0140-6736(15)61205-3

[5] International Council for Science ; Griggs DJ, Nilsson M, Stevance A, McCollum D, editors. A guide to SDG interactions: from science to implementation. Paris: ICSU https://council.science/cms/2017/05/SDGs-Guide-to-Interactions.pdf (viewed July 2018).

[6] Department of Health, Australian Government . Senate Standing Committee on Foreign Affairs, Defence and Trade Inquiry into the United Nations Sustainable Development Goals (SDGs). Department of Health submission to the inquiry. Parliament of Australia, 2018 https://www.aph.gov.au/Parliamentary_Business/Committees/Senate/Foreign_Affairs_Defence_and_Trade/SDGs/Submissions (viewed Oct 2018).

[7] Brolan CE , Gouda HN , AbouZahr C , Lopez AD . Beyond health: five global policy metaphors for civil registration and vital statistics. Lancet 2017; 389: 1084–1085. 28322806 10.1016/S0140-6736(17)30753-5

[8] United Nations Sustainable Development Knowledge Platform . Australia Voluntary National Review 2018. https://sustainabledevelopment.un.org/content/documents/20470VNR_final_approved_version.pdf (viewed July 2018).

[9] Australian Government . 2017 Foreign Policy White Paper [website]. https://www.fpwhitepaper.gov.au (viewed Jul 2018).

[10] Australian Institute of Health and Welfare . Australia's health 2018 (AIHW Cat. No. AUS 221). Canberra: AIHW, 2018 https://www.aihw.gov.au/reports/australias-health/australias-health-2018/contents/table-of-contents (viewed July 2018).

[11] National Congress of Australia's First Peoples . Submission to the Senate Foreign Affairs, Defence and Trade References Committee; May 2018. Parliament of Australia, 2018. https://www.aph.gov.au/Parliamentary_Business/Committees/Senate/Foreign_Affairs_Defence_and_Trade/SDGs/Submissions (viewed Nov 2018).

[12] Anderson I , Robson B , Connolly M , et al. Indigenous and tribal peoples’ health (*The Lancet*–Lowitja Institute Global Collaboration): a population study. Lancet 2016; 388: 131–157. 27108232 10.1016/S0140-6736(16)00345-7

[13] Shannon C , Brolan CE , Gajjar D . Realising the potential of the post‐2015 development agenda for Indigenous health. Med J Aust 2014; 201: 202 https://www.mja.com.au/journal/2014/201/4/realising-potential-post-2015-development-agenda-indigenous-health 25164844 10.5694/mja14.00059

[14] Green D , King U , Morrison J . Disproportionate burdens: the multidimensional impacts of climate change on the health of Indigenous Australians. Med J Aust 2009; 190: 4–5. https://www.mja.com.au/journal/2009/190/1/disproportionate-burdens-multidimensional-impacts-climate-change-health 19119999 10.5694/j.1326-5377.2009.tb02250.x

[15] Department of Foreign Affairs and Trade, Australian Government . Senate Foreign Affairs, Defence and Trade References Committee. Inquiry into the United Nations Sustainable Development Goals. Department of Foreign Affairs and Trade Submission; March 2018. https://www.aph.gov.au/Parliamentary_Business/Committees/Senate/Foreign_Affairs_Defence_and_Trade/SDGs/Submissions (viewed Nov 2018).

[16] Brolan C . Australia needs political courage, will and leadership to realise the SDGs. Devpolicy Blog 2018; 17 Oct. http://www.devpolicy.org/australia-needs-political-courage-will-leadership-to-realise-sdgs-20181017 (viewed Nov 2018).

[17] Sachs J , Schmidt‐Traub G , Kroll C , et al. SDG Index and Dashboards report 2018. New York: Bertelsmann Stiftung and Sustainable Development SolutionsNetwork, 2018. http://sdgindex.org/reports/2018/ (viewed Nov 2018).

