# Supplementary material for: Health's role in achieving Australia's Sustainable Development Goal commitments
Source: Med J Aust. 2019 Feb 24;210(5):204–206.e1. doi: 10.5694/mja2.50040 (PMC6850417; doi:10.5694/mja2.50040)
Supplement: Supplementary file 1 [file MJA2-210-204-s001.pdf]

MJA
The Medical Journal of Australia

Supporting Information

Supplementary figures and tables

This appendix was part of the submitted manuscript and has been peer reviewed.
It is posted as supplied by the authors.

Appendix to: Brolan CE, Hall N, Creamer S, et al. Health's role in achieving Australia's Sustainable Development Goal commitments. *Med J Aust* 2019; doi: 10.5694/mja2.50040.

Figure 1. International Council for Sciences demonstrates how Sustainable Development Goal (SDG) 3 interacts with the other SDGs[1]

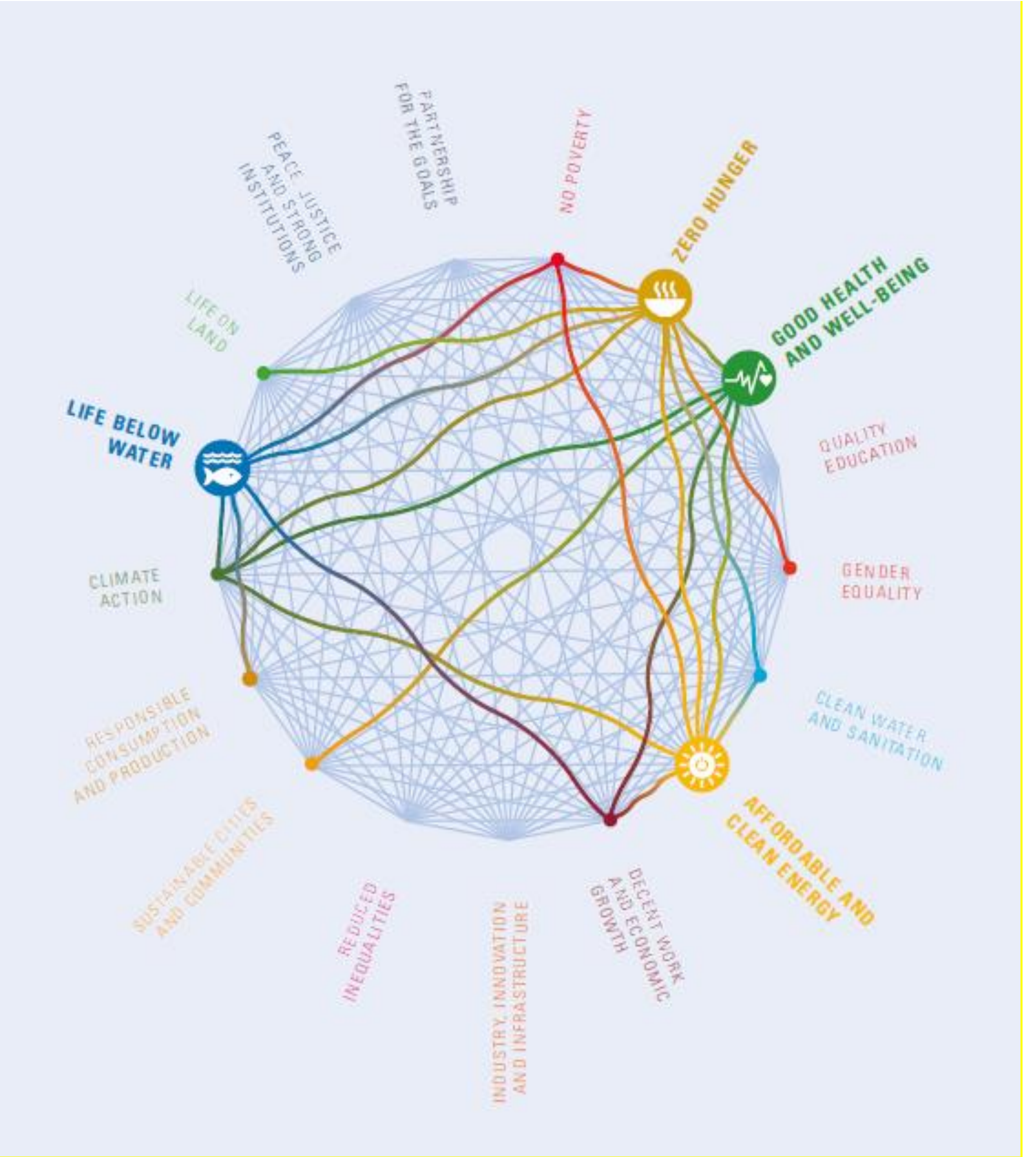

Table 1. Sustainable Development Goal (SDG) implementation in Australia – a snapshot and timeline of government and non-government activities

Date	Event
September 2015	▪ Australia, along with 192 UN Member States, commits to implement the 2030 Agenda for Sustainable Development and its 17 Sustainable Development Goals (SDGs)
April 2016	▪ Formation of the UN High Level Panel on Water, to which Australia is one of the 11 country members
September 2016	▪ Inaugural Australian SDG Summit, first high-level multi-stakeholder forum to advance national implementation of the SDGs
October 2016	▪ National Youth Summit on the SDGs, Melbourne
November 2017	▪ Second Sustainable Development Solutions Network (SDSN) and Australian Council for International Development (AFCID) high-level multi-stakeholder SDG Summit, Sydney
July 2017	▪ Australia's Ambassador to the UN announced Australia would complete its first Voluntary National Review (VNR) on the SDGs at the UN's High Level Political Forum (HLPF) in July 2018
November 2017	▪ Australian Department of Health ran online stakeholder engagement process seeking input into Australia's VNR<br>▪ Australian Government releases its 2017 Foreign Policy White Paper, and commits to working with partners to achieve the SDG agenda in its development program
December 2017	▪ The Australian Senate referred the matter of the "UN SDGs" to the Foreign Affairs, Defence and Trade (DFAT) References Committee for Inquiry
January 2018	▪ Australian Department of Health closes its online VNR stakeholder engagement process
March 2018	▪ Third multi-stakeholder SDG summit, Melbourne<br>▪ Public submissions to the Parliamentary Inquiry into the UN SDGs close
March 2018	▪ UN High Level Panel on Water mandate ended with the release of its outcome package
June 2018	▪ Australia releases its first VNR
July 2018	▪ Australia presents its VNR on SDG progress to the HLPF on Sustainable Development in New York
November 2018	▪ Anticipated release-date of the DFAT report on the Parliamentary Inquiry into the "UN SDGs"

Table 2. Summary of the Australian Government's Voluntary National Review content on Sustainable Development Goal 3

Areas for Australian action	Action points	Australian populations in focus
Access to a world-class healthcare system (including primary health care) that can effectively and quickly respond to emerging threats, potential disease outbreaks and disaster	▪ Universal health care coverage (Medicare, Pharmaceutical Benefits Scheme)<br>▪ Support for hospitals<br>▪ Prioritising preventive health (to tackle NCD risk factors and its burden) and mental health programs and supports<br>▪ Investment in public health and medical research<br>▪ Australian governments collaborate with CSOs and health providers to optimally address healthcare gaps	▪ Aboriginal and Torres Strait Islander Peoples<br>▪ Lesbian, Gay, Bisexual, Transgender, Queer, Intersex and other (LGBTQI) communities<br>▪ Regional, rural and remote populations<br>▪ Australians experiencing chronic disease<br>▪ Australians living with mental health issues
Addressing Aboriginal and Torres Strait Islander Peoples' health care	▪ Implementing the National Aboriginal and Torres Strait Islander Health Plan by integrating Indigenous concepts of health and well-being<br>▪ Cross-jurisdiction collaboration with Aboriginal and Torres Strait Islander organisations for improved Indigenous health<br>▪ Supporting the Australian Model of First 1000 Days, which focuses on reducing under-nutrition in Aboriginal and Torres Strait Islander families	▪ Aboriginal and Torres Strait Islander Peoples
Access to regional, rural and remote health services	▪ Innovation and technology - transition towards greater integration of digital technology including mobile health, online heath records and telehealth systems<br>▪ Redress unequal distribution of health professionals between inner-metropolitan and rural and remote communities<br>▪ Role of National Rural Health Commissioner	▪ Regional, rural and remote populations

Table 2: Australian Government agencies for domestic reporting on the Sustainable Development Goals for the Voluntary National Review[2]

Goal		Lead Agency	Supporting Agencies
1	End poverty in all its forms everywhere	Social Services	▪ Prime Minister and Cabinet<br>▪ Australian Bureau of Statistics<br>▪ Home Affairs
2	End hunger, achieve food security and improved nutrition and promote sustainable agriculture	Agriculture and Water Resources	▪ Health
3	Ensure healthy lives and promote well-being for all at all ages	Health	
4	Ensure inclusive and equitable quality education and promote lifelong learning opportunities for all	Education and Training	
5	Achieve gender equality and empower all women and girls	Prime Minister and Cabinet	▪ Social Services
6	Ensure availability and sustainable management of water and sanitation for all	Agriculture and Water Resources	▪ Environment and Energy
7	Ensure access to affordable, reliable, sustainable and modern energy for all	Environment and Energy	▪ Industry, Innovation and Science
8	Promote sustained, inclusive and sustainable economic growth, full and productive employment and decent work for all	Treasury	▪ Jobs and Small Business<br>▪ Australian Bureau of Statistics
9	Build resilient infrastructure, promote inclusive and sustainable industrialisation and foster innovation	Infrastructure, Regional Development and Cities	▪ Industry, Innovation and Science<br>▪ Communications and the Arts
10	Reduce inequality within and among countries	Treasury	▪ Social Services<br>▪ Home Affairs
11	Make cities and human settlements inclusive, safe, resilient and sustainable	Infrastructure, Regional Development and Cities	▪ Communications and the Arts<br>▪ Home Affairs
12	Ensure sustainable consumption and production patterns	Environment and Energy	▪ Agriculture and Water Resources<br>▪ Finance
13	Take urgent action to combat climate change and its impacts	Environment and Energy	▪ Home Affairs
14	Conserve and sustainably use the oceans, seas and marine resources for sustainable development	Environment and Energy	▪ Agriculture and Water Resources<br>▪ Home Affairs (Maritime Border Command)<br>▪ Infrastructure Regional Development and Cities (Australian Maritime Safety Authority)
15	Protect, restore and promote sustainable use of terrestrial ecosystems, sustainably manage forests, combat desertification, and half and reverse land degradation and halt biodiversity loss	Environment and Energy	▪ Agriculture and Water Resources
16	Promote peaceful and inclusive societies for sustainable development, provide access to justice for all and build effective, accountable and inclusive institutions at all levels	Attorney-General	▪ Defence
17	Strengthen the means of implementation and revitalise the global partnership for sustainable development	Department of Foreign Affairs and Trade	▪ Treasury<br>▪ Australian Bureau of Statistics

References

1. International Council for Science; Griggs DJ, Nilsson M, Stevance A, McCollum D, editors. A guide to SDG interactions: from science to implementation. Paris: ICSU https://council.science/cms/2017/05/SDGs-Guide-to-Interactions.pdf (viewed July 2018).
2. Department of Foreign Affairs and Trade, Australian Government. Senate Foreign Affairs, Defence and Trade References Committee. Inquiry into the United Nations Sustainable Development Goals. Department of Foreign Affairs and Trade Submission; March 2018. https://www.aph.gov.au/Parliamentary_Business/Committees/Senate/Foreign_Affairs_Defence_and_Trade/SDGs/Submissions (viewed Nov 2018).
